# Exploratory study on the safety and effectiveness of Yizhi Qingxin Decoction (capsules) in the treatment of hypertension in the elderly with mild cognitive impairment (deficiency of kidney essence syndrome)

**DOI:** 10.1097/MD.0000000000020789

**Published:** 2020-07-02

**Authors:** Bi-Qing Wang, Jun Mei, Lu Liu, Chun-Xiao Ju, Jun-Nan Zhao, Ping Zhang, Feng-Qin Xu, Ke-Ji Chen

**Affiliations:** a Clinical College, Beijing University of Chinese Medicine; b Xiyuan Hospital, China Academy of Chinese Medical Sciences; c Institute of Geriatrics; d Cardiovascular Diseases Center, Xiyuan Hospital, China Academy of Chinese Medical Sciences, Beijing, China.

**Keywords:** Yizhi Qingxin Decoction, hypertension, mild cognitive impairment, traditional Chinese medicine

## Abstract

**Background:**

Hypertension in the elderly with cognitive impairment has been one of the global health issues. Mild cognitive impairment (MCI) is the state of transition between the normal aging process and cognitive changes of unformed dementia. Diagnosis and treatment of MCI are the keys to prevent dementia, and hypertension is one of the important influencing factors of MCI. Our preclinical experiment found that Yizhi Qingxin Decoction (YQD) could effectively reduce the blood pressure of spontaneously hypertensive rats (SHR), improve their spatial learning and memory abilities in Morris water maze, and play a neuroprotective role. The objective is to estimate the safety and efficacy of YQD (capsules) in the treatment of hypertension in the elderly with MCI (deficiency of kidney essence syndrome) through this study.

**Methods:**

According to the random number generated by the block random method, 100 participants will be randomly and equally divided into the treatment group (YQD) or the control group (*Ginkgo biloba* extract tablets). The conversion rate of dementia will be used as the main evaluating indicator by the CDR scale. The MoCA scale, MMSE scale, ADCS-MCI-ADL-24 scale, CGIC-KDS scale, and 24-h ambulatory blood pressure will be used as the secondary evaluating indicator. Safety will be evaluated based on specific manifestations of adverse reactions and the incidence of adverse events.

**Objective:**

The objective is to estimate the curative effect of YQD (capsules) on hypertension in the elderly with MCI (deficiency of kidney essence syndrome), and to evaluate the safety of its clinical application.

**Trial registration:**

Chinese Clinical Trial Registry (ICTRP member): ChiCTR2000030292.

## 1 Introduction

As the population ages, the burden of hypertension on the global population is increasing,^[1]^ the cost of health care increases due to low quality of life affected by cognitive impairment or dementia. Hypertension in the elderly with cognitive impairment has been one of the global public health problems. The prevalence of hypertension in the elderly increased linearly, with more than 70% of the population over 70 years old.^[2]^ Mild cognitive impairment (MCI) is a transitional state between the normal aging process and cognitive changes in early dementia, and the incidence of MCI in people over 70 years old is nearly 20%.^[3]^ Current studies have shown that hypertension is correlated with the occurrence and development of cognitive impairment and dementia.^[4]^ The risk of MCI and dementia can be reduced by keeping blood pressure within the appropriate range.^[5]^

The latest 2017 ANN guideline for MCI makes clear that there is no high-quality evidence to support drug therapy for MCI.^[3]^ Preventing risk factors without the use of cognitive impairment drugs is the first step in controlling MCI. *Ginkgo biloba* preparations have also not been shown to reduce cognitive decline in the elderly with normal or MCI. A trial with an average follow-up of 6.1 years abroad, surveyed 3069 community residents, taking 120 mg of *Ginkgo biloba* extract tablets (Ginaton) twice daily, with the placebo of the same appearance. In comparison, it showed no significant difference in the incidence of adverse events.^[6]^ But research shows that *Ginkgo biloba* extract has a protective effect on the decline of memory.^[7]^ Some in vitro studies have shown that *Ginkgo biloba* extract may inhibit the aggregation of amyloid protein, suggesting another mechanism to prevent or delay the cognitive decline associated with Alzheimer's disease.^[8]^ In summary, *Ginkgo biloba* extract may prevent or delay age-related changes in individuals with normal cognitive abilities, or slow down the cognitive decline in patients with MCI, and *Ginkgo biloba* extract tablets (Ginaton) have not found evidence of long-term use to increase the incidence of adverse events.

In traditional Chinese medicine, MCI can be classified into the category of “amnesia” and “dementia.” At present, it is believed that the deficiency of kidney essence is the fundamental factor of MCI. Wind, fire, phlegm, and blood stasis are the key factors for the development of the disease. Academician Ke-Ji Chen summarized Yizhi Qingxin Decoction (YQD) based on his many years of clinical experience in MCI treatment of elderly hypertension. The composition of the formula is *Alpinia oxyphylla Miq*, *Lycii Fructus*, and so on. Its efficacy is mainly based on tonifying the kidney and replenishing essence, as well as clearing the heart and liver heat.

Our preclinical experiment found that YQD could effectively reduce the blood pressure of spontaneously hypertensive rats (SHR), improve their spatial learning and memory abilities in Morris water maze, and play a neuroprotective role. It can inhibit aortic fibrosis and reduce serum aldosterone and angiotensin II levels in SHR. In this trial, *Ginkgo biloba* extract tablets (Ginaton) will be used as a positive control drug to estimate the efficacy of YQD (capsules) on hypertension in the elderly with MCI (deficiency of kidney essence syndrome) and the safety of its clinical application.

## 2 Clinical trial design and protocol

This trial has been registered in the Chinese Clinical Trial Registry and meets the specification of the CIOMS international ethical guidelines for human biomedical research. The results will be reported in accordance with the CONSORT extension recommendation of the 2017 Chinese herbal formula test report.^[9]^

**Figure 1:**
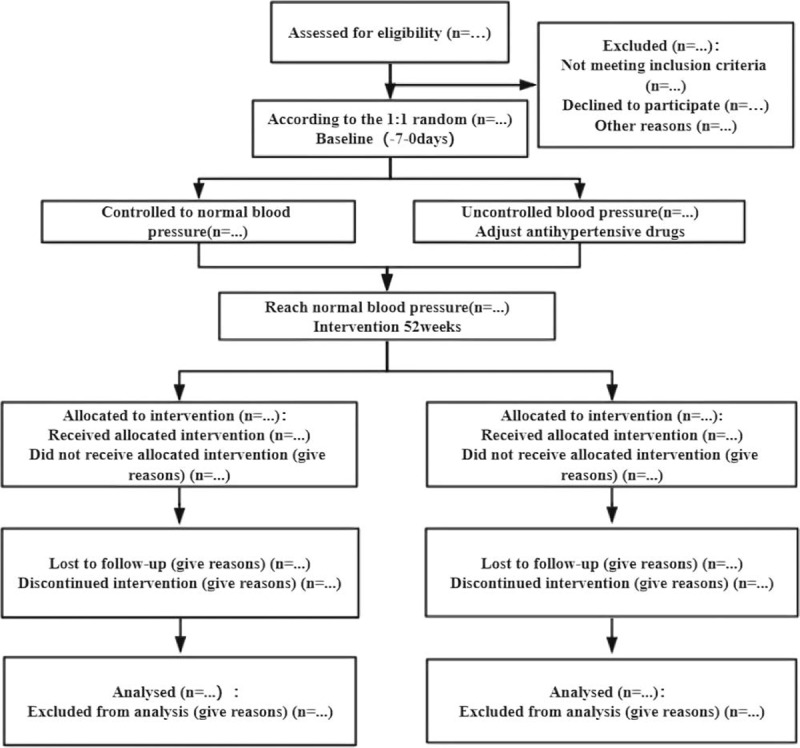
Flow chart of the study.

The study uses a positive-drug parallel randomized control design. Participants will fully understand the benefits and risks and sign a written informed consent before being included in this study. The personal data of participants will be kept strictly by the researchers in order to protect privacy. The enrolled participants (n = 100) are randomly divided into a treatment group (n = 50) and a control group (n = 50). The two groups of participants will be measured for 24 h ambulatory blood pressure. If the blood pressure is not within the normal range, we will adjust the use of antihypertensive drugs, and check it 1 month later to make all patients reach the standard blood pressure. Both groups will receive 52 weeks of treatment and 5 follow-ups, as shown in Figure 1.

### 2.1 Source of cases

Researchers will recruit 100 participants through advertising, outpatient, etc. A total of 100 elderly participants who meet the inclusion and fail to meet exclusion criteria will be enrolled.

### 2.2 Diagnostic criteria

#### 2.2.1 Diagnostic criteria for hypertension in the elderly

Refer to the revised Guideline for the prevention and treatment of hypertension in China,^[10]^ hypertension in the elderly diagnostic criteria: The sitting systolic blood pressure (SBP) ≥140 mm Hg and/or diastolic blood pressure (DBP) ≥90 mm Hg measured three times in different days among patients aged ≥65 years or older.

#### 2.2.2 Diagnostic criteria for MCI

Refer to China's Guidelines for MCI,^[11]^ MCI diagnostic criteria: Report by patients or informed individuals, or experienced clinicians discover cognitive impairment; objective evidence of impairment of one or more cognitive functional domains (from cognitive tests); complex instrumentality daily abilities can be slightly impaired, but the independent daily living ability is maintained; the disease has not yet reached the diagnosis of dementia.

Auxiliary diagnostic criteria: The MoCA scale score <26 points; the MMSE scale score is graded according to the educational level, with 21 points ≤ primary school ≤26 points, and 24 points ≤ middle school or above (including technical secondary school) ≤ 26 points; the CDR scale score is 0 or 0.5 points.

#### 2.2.3 Traditional Chinese medicine syndrome diagnosis criteria

Refer to the Kidney Deficiency Syndrome Scale dialectical standard developed by Jin-Zhou Tian in 2017 (Table 1),^[12]^ the sum of all the symptom scores is ≥7, which meets the diagnostic criteria of kidney deficiency syndrome.

**Table 1:**
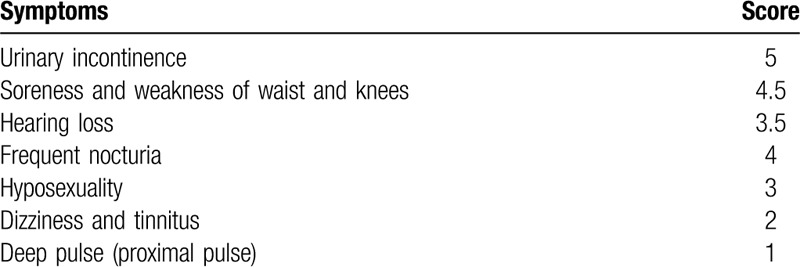
Kidney deficiency syndrome.

### 2.3 Inclusion and exclusion criteria

#### 2.3.1 Inclusion criteria

- Meet the diagnostic criteria of hypertension in the elderly.
- Meet MCI diagnostic criteria.
- Meet the Kidney Deficiency Syndrome Scale dialectical standard.
- Aged between 65 and 75 years old.
- Adequate vision and audiology for neuropsychological testing.
- Have a certain level of education, can read and write simple sentences.
- Have a signed informed consent form.

#### 2.3.2 Exclusion criteria

- Dementias of various causes that have met the DSM-IV diagnostic criteria for dementia, including Alzheimer's disease, vascular dementia, and other types of dementia.
- Sleep disorders that may affect cognitive function, drug dependence, or alcohol dependence in the previous 5 years, and other causes that may interfere with cognitive performance, for example, depressive disorders (HAMD > 12) or mental disorders.
- CT or MRI scans within the past 12 months revealed central nervous system infection, infarction, or other focal lesions; Hachinski Ischemic Scale (HIS) > 4.
- Other conditions are not suitable for participation, such as hypertensive emergencies, hypertensive crises, high blood pressure that has not been controlled despite medication, severe arrhythmia, the patients who occurred myocardial infarction within 3 months before participating in this trial.
- Patients who had regularly taken traditional Chinese medicines with similar effects, or anticholinergic drugs, anticonvulsants, antiparkinsonian, excitatory drugs, cholinergic drugs, antipsychotics, anti-anxiety drugs, or who were known to be allergic to the trial drugs before this trial.
- Severe liver and kidney dysfunction; severe asthma and chronic obstructive pulmonary disease; severe digestive system disease.
- Participants are participating in other clinical trials.
- Participants are deemed unable to follow the trial procedures.

#### 2.3.3 Abort/exit criteria

- Some participants’ condition constantly worsens or the participants emerge severe conditions during the trial such as acute hypertension, acute cerebral infarction, intracerebral hemorrhage, deterioration of cardiac function, acute coronary syndrome. The participant will withdraw from the trial.
- Some participants with cognitive function decline during the trial, will continue to take medication and be followed up. If the participant's condition turns into dementia, the participant will be transferred to conventional treatment, and this case will be terminated.
- A case judged by a doctor as a possible dangerous event should be stopped. To protect the participant, the participant should withdraw from the clinical trial and receive other treatments. The case is treated as invalid.
- If some participants emerge certain comorbidities or special physiological changes during the trial, they will not be appropriate to continue the trial.
- If some participants could not follow the clinical trial protocol in terms of taking drugs, receiving visits, and so on during the trial, they will be considered to discontinue participating in the trial because they may affect the authenticity of the study results.
- Participants with adverse events or severe adverse events.

After the termination of the case, the case record form should be kept, and the final test results should be taken as the final results to analyze the efficacy and adverse reaction data.

### 2.4 Intervening measures

#### 2.4.1 Basic measures

Basic measures in the two groups will be included improvement of lifestyle to eliminate adverse factors for physical and mental health, such as weight control, reduction of dietary fat intake, appropriate restriction of salt, maintenance of exercise.

#### 2.4.2 Drugs intervention

Treatment group: YQD (capsules) 0.4 g/capsule, 3 times a day, 4 capsules once a time, oral after meals.

Control group: *Ginkgo biloba* extract tablets (Ginaton) 40 mg/tablet, 2 times a day, 2 tablets once a time, oral after meals.

#### 2.4.3 Group enrollment and follow-up

Both participants will be measured for 24 h ambulatory blood pressure when they enter the group. If they do not reach the normal blood pressure range, their antihypertensive drugs will be adjusted. After 1 month, the blood pressure will be reviewed to make all participants reach the standard blood pressure. The course of treatment is 52 weeks, and 5 follow-ups are set up, at 4 weeks, 12 weeks, 24 weeks, 36 weeks, and 52 weeks.

#### 2.4.4 Combined medication

- During the trial, it is forbidden to add Chinese and western medicines that have the same or similar effects or affect the safety evaluation of the trial drugs, including drugs that improve cognitive function such as Compound Congrong Yizhi Capsule, other *Ginkgo biloba* preparations, similar Chinese medicine or decoction, aniracetam, nicergoline. If combination therapy is required, the participant should be treated as an excluded case. The name of the combined medication used, the total daily dosage, the reason for taking it, and the start and end time of taking it should be recorded at the time of follow-up.
- Combination therapy that must be taken for other diseases, can continue to be taken, and record name, dosage, and the number of times in the medical record for summary and report.

### 2.5 Outcome indicators

#### 2.5.1 Main efficacy indicators

- Conversion rate of dementia: The CDR scale.
- Efficacy determination criteria: The follow-up test CDR scale score is 0 or 0.5, which has not converted to dementia and reaches the main efficacy indicators.

#### 2.5.2 Secondary evaluation indicators

- Cognitive function: MoCA scale, the MMSE scale is graded according to educational level.
- Daily life ability: MCI daily living ability scale (ADCS-MCI-ADL-24).
- 24-hour average blood pressure: day-average blood pressure, night-average blood pressure, 24 h blood pressure variability.
- Clinical Global Impression of Change-Kidney Deficiency Syndrome (CGIC-KDS): Changes in syndrome overall impression based on physician experience and caregiver supplementation.

#### 2.5.3 Safety indicators

- All adverse events.
- General physical examination.
- Other tests: Blood test, urine test, fasting blood glucose, biochemical assay, electrocardiogram.

### 2.6 Study period

The study period based on SPIRIT 2013 statement^[13]^ (Table 2).

**Table 2:**
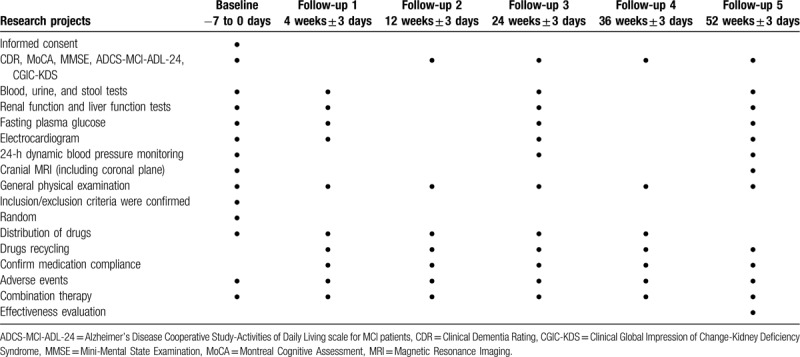
Study period.

### 2.7 Sample size calculation

There is currently a lack of published clinical data on the experimental drugs for sample size calculations. We designed the small sample size study of 100 participants to preliminarily estimate the efficacy of YQD (capsules) for the treatment of hypertension in the elderly with MCI.

### 2.8 Management and treatment of adverse events

Expected adverse events include gastrointestinal reactions, such as abdominal discomfort, nausea or vomiting; bronchial allergic reactions, for instance, dyspnea, asthma; skin allergies, for example, rash, urticaria, pruritus; liver and kidney function abnormalities (>2 times of normal value). The participants will be advised to stop taking the trial drugs when the above adverse events occur. All adverse events except the expected adverse events are treated as unexpected adverse events, recorded and reported by the adverse events reporting requirements, and timely and appropriate medical treatment by the clinical diagnosis and treatment norms.

If serious adverse events occur during the trial, regardless of whether they are related to the trial drugs, we will give timely rescue treatment to ensure the safety of the participants and give an account of the ethics committee within 24 h or not later than the second working day. We will sign the report and indicate the date and complete a “serious adverse event report form,” which records the time, place, and person of the report. All adverse events will be traced to normal or nearly normal to ensure the safety of the participants.

### 2.9 Quality control

All researchers can enter and conduct this trial only after finishing training and authorization from the principal researcher. The person in charge shall conduct Good Clinical Practice (GCP) and protocol training for all researchers and other persons related to the trial.

### 2.10 Random method and assignment concealment

The block random method is adopted to enhance the equilibrium comparability between the two groups. The random code table is generated by Statistical Analysis System (SAS) software. It is divided into 2, one is stored in the designated computer in the form of the electronic document. The other one is kept in an opaque envelope for hidden distribution. The drug administrators will issue drugs according to the order of each participant's medical treatment and drug number.

### 2.11 Data management

The random sequence is generated by the data administrator and is unavailable to the researchers who will recruit participants. Data administrator will be performed by two data entry personnel independently for double entry and proofreading. They will randomly select a certain number of case report forms and compare them with the data in the database to ensure that the data is correct.

### 2.12 Statistical analysis

Statistical Product and Service Solutions (SPSS24.0) software will be used for statistical analysis of data. The different data types are analyzed by appropriate methods. Per-protocol set (PPS) and Full analysis set (FAS) will be implemented simultaneously for the main outcome indicators. All randomized patients who received at least one treatment and completed the post medication safety evaluation will use the safety set (SS) to evaluate the safety. The least-square mean (LSMEAN) of analysis of covariance and its 95% confidence limit, or logistic regression will determine the difference in efficacy between groups and eliminate the influence of confounding factors on efficacy. The statistical test is conducted by a bilateral difference test, if *P* is <.05, the data difference is significant.

## 3 Discussion

The objective of this study is to estimate the safety and efficacy of YQD (capsules) in the treatment of hypertension in the elderly with MCI (deficiency of kidney essence syndrome). The prescription composition of YQD includes *Alpinia oxyphylla Miq*, *Lycii Fructus*, etc. YQD has the function of tonifying kidney essence. The deficiency of kidney essence is the fundamental factor of MCI in the theory of traditional Chinese medicine. A modern medical research has found that *5-HMF*, the main active component of *Alpinia oxyphylla Miq*, can improve the memory impairment of mice induced by *Aβ*_*1–42*_, which has a potential therapeutic effect on dementia.^[14]^*Lycii Fructus* extract can improve the cognitive function in mice of Alzheimer's disease, reduce *Aβ* deposition, and regulate the expression of brain neurotrophic derived factor and tyrosine kinase *B*.^[15]^

MCI is considered a clinical syndrome with impaired participative and objective memory or other cognitive functions, but the function of daily life remains basically intact.^[16]^ The prevalence of MCI in the elderly population (older than 65 years) ranges from 3% to 19%, and the conversion rate of dementia within 2 years ranges from 11% to 33%.^[17]^ The treatment of MCI falls under the category of secondary prevention of dementia. The treatment goal of MCI is to improve symptoms, and it is more important to delay or prevent further damage in cognitive ability and the onset of dementia. It is helpful to evaluate the therapeutic effect of drugs based on the outcome indicators of the conversion rate of dementia and cognitive function.^[3]^ The self-regulating imbalance of cerebral blood flow and cerebral perfusion caused by long-term hypertension is the main biological pathway of hypertension with cognitive impairment. Duration of hypertension could be a determining factor of cognitive decline in the elderly.^[5,18]^ Early control of blood pressure is beneficial to reduce cognitive decline. The studies have shown that the increase of blood pressure variability may be related to the decline of cognitive function, which still exists in patients with good blood pressure control.^[19–21]^ 24-h ambulatory blood pressure monitoring can effectively provide information about blood pressure changes.

The study is a positive-drug parallel randomized controlled trial, using a block randomization method to enhance the equilibrium comparability between two groups. Assignment concealment reduces selection bias. According to the inclusion and exclusion criteria, the homogeneity of research objects is improved. In the treatment group, YQD will be made into capsules to increase the compliance and convenience of elderly participants. The conversion rate of dementia (CDR scale) is the main efficacy indicator. The MMSE scale, MOCA scale, ADCS-MCI-ADL-24 scale, CGIC-KDS scale, and 24-h ambulatory blood pressure observation are the secondary efficacy indicators, to provide the certain reference for the efficacy evaluation of outcome. The treatment duration is 52 weeks, include 5 follow-ups during the treatment period, providing reliable results for the evaluation of prognosis and observation of adverse reactions.

The methodological limitation includes failing to achieve double-blind. And this is a single-center study, with a small sample size and relatively short time for people with MCI. Therefore, there is a lack of long-term evaluation of the main outcome indicators (conversion rate of dementia), which limits the efficacy evaluation of drugs. In the future, it is necessary to carry out a multicenter randomized double-blind study to reduce methodological bias and conclusion deviation, and extend the follow-up time to further evaluate the effect of YQD on dementia prevention and cognitive function.

## 4 Others

The study was accepted by the ethics committee of Xiyuan Hospital, Chinese Academy of Medical Sciences in February 2020 (approval NO. 2020XLA005-2). The study will be based on the requirements of the Helsinki Declaration of the World Medical Association. Currently, the researchers are recruiting participants.

## Acknowledgments

The authors sincerely thank all the participants and the researchers who will participate in the trial for their assistance.

## Author contributions

KJC proposed the concept, PZ and FQX designed the trial, PZ, JM, and BQW were responsible for data collation, FQX conducted research supervision, BQW, JM, LL, and CXJ wrote the first draft, PZ, JM, and JNZ reviewed and checked the manuscript.

**Conceptualization:** Ke-Ji Chen.

**Investigation:** Bi-Qing Wang, Jun Mei, Ping Zhang.

**Methodology:** Ping Zhang, Feng-Qin Xu.

**Project administration:** Feng-Qin Xu.

**Writing – original draft:** Bi-Qing Wang, Lu Liu, Chun-Xiao Ju.

**Writing – review & editing:** Jun Mei, Jun-Nan Zhao, Ping Zhang.
